# Heterogeneity of Red Blood Cells: Causes and Consequences

**DOI:** 10.3389/fphys.2020.00392

**Published:** 2020-05-07

**Authors:** Anna Bogdanova, Lars Kaestner, Greta Simionato, Amittha Wickrema, Asya Makhro

**Affiliations:** ^1^Red Blood Cell Research Group, Vetsuisse Faculty, The Zurich Center for Integrative Human Physiology (ZHIP), Institute of Veterinary Physiology, University of Zurich, Zurich, Switzerland; ^2^Experimental Physics, Dynamics of Fluids, Faculty of Natural Sciences and Technology, Saarland University, Saarbrücken, Germany; ^3^Theoretical Medicine and Biosciences, Medical Faculty, Saarland University, Homburg, Germany; ^4^Institute for Clinical and Experimental Surgery, Saarland University, Homburg, Germany; ^5^Section of Hematology/Oncology, Department of Medicine, University of Chicago, Chicago, IL, United States

**Keywords:** red blood cells, heterogeneity, morphology, erythroid precursor cells, age

## Abstract

Mean values of hematological parameters are currently used in the clinical laboratory settings to characterize red blood cell properties. Those include red blood cell indices, osmotic fragility test, eosin 5-maleimide (EMA) test, and deformability assessment using ektacytometry to name a few. Diagnosis of hereditary red blood cell disorders is complemented by identification of mutations in distinct genes that are recognized “molecular causes of disease.” The power of these measurements is clinically well-established. However, the evidence is growing that the available information is not enough to understand the determinants of severity of diseases and heterogeneity in manifestation of pathologies such as hereditary hemolytic anemias. This review focuses on an alternative approach to assess red blood cell properties based on heterogeneity of red blood cells and characterization of fractions of cells with similar properties such as density, hydration, membrane loss, redox state, Ca^2+^ levels, and morphology. Methodological approaches to detect variance of red blood cell properties will be presented. Causes of red blood cell heterogeneity include cell age, environmental stress as well as shear and metabolic stress, and multiple other factors. Heterogeneity of red blood cell properties is also promoted by pathological conditions that are not limited to the red blood cells disorders, but inflammatory state, metabolic diseases and cancer. Therapeutic interventions such as splenectomy and transfusion as well as drug administration also impact the variance in red blood cell properties. Based on the overview of the studies in this area, the possible applications of heterogeneity in red blood cell properties as prognostic and diagnostic marker commenting on the power and selectivity of such markers are discussed.

## Introduction

Our understanding of red blood cells (RBCs) evolved from acknowledgment of the basic and fundamental role of these cells as key players in gas exchange to the state where we assign multiple complex functions related to sensing and signaling, maintenance of homeostasis of pH and redox state and participation in control of vascular tone, clotting (Andrews and Low, 1999; Bernhardt et al., 2019), and other processes (Helms et al., 2018; Pernow et al., 2019).

Broadening of RBC functions was accompanied with our awareness of complexity of the cellular architecture and biochemistry. Spatial compartmentalization of processes and resources in RBCs was discovered (Hoffman et al., 2009; Chu et al., 2012). Complex dynamics precise orchestration of processes occurring in the circulating RBCs in response to the changes in micro- and macro-environment (hormonal and mechanical stimulation, changes in local or ambient oxygen availability, temperature, circadian rhythm-related processes and others) is becoming evident (e.g., O’Neill and Reddy, 2011; Cahalan et al., 2015; Zhou et al., 2019).

With time it became clear that these changes and responses do not necessarily involve all the circulating cells. As our knowledge of these cells accumulates more and more reports mention the presence of “responding” and “non-responding” cells in the circulation (e.g., Kaestner et al., 2012; Makhro et al., 2013; Wang et al., 2013; Rotordam et al., 2019). As we recognize the existence of multiple fractions of RBCs that are functionally different from each other, we feel a growing need to unravel the nature of these differences, their causes and the potential information hidden in RBC heterogeneity on systemic distress and pathology. In this review we aimed to summarize the current state of knowledge in this rapidly developing research area. We focus on RBCs of healthy humans and give only a few examples of how RBC heterogeneity may be used to predict RBC disease nature and severity. Heterogeneity of stored or transfused RBCs is a broad topic also out of the scope of this review.

## Inter-Individual Heterogeneity

Inter-individual variation in properties of circulating RBCs of healthy donors reflects genetic and epigenetic variance as well as the state in which the organism resides over the past 3–4 months during which the cells undergo transitions from erythroid progenitors to young, mature, and senescent state. Variance spreads to the number of copies of proteins per cell, activity of enzymes and ion transporters, shapes, differences in density, deformability, membrane stability, redox state, and the collection of Hb variants in a given cell. Most of the studies for healthy humans were performed on stored blood to assess its quality and identify a cohort of best donors (Sparrow, 2017).

The possible causes of this inter-individual variation originate at the level of erythroid precursor cells (as in case of ineffective erythropoiesis (Oikonomidou and Rivella, 2018), for more details see section Cellular Heterogeneity During Erythropoiesis) or emerge later on as the cells enter the circulation and get exposed to a variety of microenvironments (osmolarity gradients in the kidneys, shear in capillaries and spleen, changes in oxygen availability and pH within peripheral tissues, changes in redox state next to the inflammatory side or to the exercising muscle). Development of heterogeneous RBC populations may be an intrinsic property of blood (e.g., RBC aging), or be triggered by the changes in life style or environmental conditions (e.g., hypoxia, microgravity) or state of the organism (e.g., stress, inflammation, changes in dietary preferences and blood metabolites). Finally, it may result from hereditary diseases that destabilize the RBC membrane or perturb its rheological properties, redox or metabolic state. In this review we focus on the possible physiological causes of heterogeneity.

## Parameters Showing Inter-Cellular Heterogeneity and Methods to Detect Them

Table 1 summarizes the information on the parameters displaying inter-individual and inter-cellular heterogeneity, as well as methodological approaches for detection of heterogeneity.

**TABLE 1:** Overview of parameters showing inter-cellular heterogeneity as well as basic principle and methodological approaches of their detection in single cells and sub-populations.

Parameter	Indicator	Method	References
Shape/size	**Direct:** Shape classification Projected area Perimeter/roughness Sphericity/elongation	Microscopy: Blood smears, images of living cells (snapshots, time series in flow, microfluidics), Imaging flow cytometers	Gonzalez-Hidalgo et al., 2015; Quint et al., 2018; Herold-Garcia and Fernandes, 2019
	Volume	Confocal microscopy + 3D deconvolution	Sadafi et al., 2019
		Scanning probe microscopy (semi quantitative)	Kihm et al., 2018
	**Indirect:** Forward and side light scatter Impedance (coulter principle)	Flow cytometry Coulter counters Multiple Blood analyzers	
Density	**Direct:** Separation according to RBC density	Fractionation in Percoll-, Stractan or similar density gradients	Lutz et al., 1992
		Lab-on-a-chip approaches	Catarino et al., 2019
	**Indirect**: Swelling- or shrinkage- resistance (e.g., the changes in SS and FS within the swelling test) Single cell rheology	Flow cytometry Cell-flow properties analyzer	Kaul et al., 2008; Barshtein et al., 2016; Fermo et al., 2017
	Membrane surface/EMA test	Flow cytometry	
Free Ca^2+^/channel activity	Fluorescent dyes for Ca^2+^ Detection of ionic currents across the membranes of single cells	Flow cytometry and fluorescence microscopy Patch-clamp incl. automated planar chips	Kaestner et al., 2006; Makhro et al., 2013; Wang et al., 2013; Fermo et al., 2017; Rotordam et al., 2019
Redox state and metabolism	Fluorescent dyes for reduced thiols (e.g., thiol tracker, monobromobimane), Fluorescent dyes for N_2_O_3_ (DAF-DA), Dyes for detection of H_2_O_2_, ONOO, HO* (e.g., H_2_DCF-DA) Single cell metabolomics (not yet used for red blood cells)	Flow cytometry Fluorescence microscopy Mass-spectrometry	Jemaa et al., 2017; Gilmore et al., 2019
Hb levels and variance	Antibodies with fluorescent tags Chromicity Sodium metabisulfite (Na_2_S_2_O5) and similar deoxygenation-based sickling tests Hemoglobin Distribution Width (HDW)	Flow cytometry, Fluorescence microscopy Microscopy	Kunicka et al., 2001; Darrow et al., 2016; Jung et al., 2016
Age	Labeling of cells (biotin conjugated with fluorescent tag or staining with PKH dyes) Reticulocyte count RNA-positive or Transferrin receptor-positive	Flow cytometry, microscopy	Mock et al., 1999; Piva et al., 2010

### Shape and Size

First descriptions of RBCs as “red corpuscles” given by Jan Swammerdam and dates back to 1658 (Swammerdam, 1737; Bessis and Delpech, 1981; Hajdu, 2003). Since then, substantial progress was made in imaging equipment as well as in fixation and staining of RBC. Blood smears still remain a part of common diagnostic practice in most of the clinical laboratories (Bain, 2005) despite the fact that smear preparation results in distortions of RBC morphology and lysis of the most fragile of them (Wenk, 1976). This technique allows to discriminate between numerous shapes from discocytes to a broad variety of “static” shapes such as echinocytes and stomatocytes, for healthy humans. The list of shape types will extend manifolds for patients with hereditary or acute disorders.

The biggest drawback of the whole approach with smears is that it provides an immediate snapshot of the shape distribution, whereas living RBCs are very dynamic entities. So are their shapes, and, rather than discussing their “absolute shape,” it would be feasible to assign them a probability to be observed in one of the shape types. The first attempts to address RBC shapes in terms of probability density distribution are recently undertaken (Reichel et al., 2019). Each cell has its “static shape” that is preferred over the other ones if no force is applied to it. There are also several preferred shape types caused by shear stress in flow. The probability to observe one of those depends on the shear rates and flow dynamics (Abkarian et al., 2008; Dupire et al., 2015; Lanotte et al., 2016; Kihm et al., 2018; Mauer et al., 2018; Reichel et al., 2019). The restoration of the initial shape of the cells as soon as the flow stops got the name of “shape memory” (Fischer, 2004; Cordasco and Bagchi, 2017). Acute shape changes associated with the ion movements across the cell membrane (dehydration or overhydration) are often reversible (Brugnara, 1997; Cossins and Gibson, 1997; Zhu et al., 2018) whereas shape alterations related to the permanent damage of the cytoskeleton or membrane loss are irreversible (Gallagher, 2005; Perrotta et al., 2008).

Preferred shapes reflect the optimal cytoskeletal conformation, hemoglobin concentration, redox state and metabolic balance and free Ca^2+^ levels that, in turn, define the activity of ion transporters, hydration state and phosphorylation state of proteins. Some of these variables will be addressed below.

Parameters to describe dynamics of RBCs morphology are currently in development. Former classifications of shapes performed by eye (Bessis and Lessin, 1970; Bessis and Delpech, 1982) are non-numerical and cannot be reliably translated into the algorithms for automated segmentation and classification of smears and images of living cells. New approaches are currently developing (Tomari et al., 2014). Roundness, roughness, projected areas are among such numeric descriptors of RBC shapes. 3D volume reconstruction of, e.g., confocal recordings are more informative than 2-dimentional images. High resolution 3D-imaging was performed for fixed RBC (Abay et al., 2019). First attempts to get the 3D imaging working for RBCs in flow are undertaken but is not yet available as a high-throughput mode (Quint et al., 2018). Cell shape recognition and classification involving artificial intelligence (AI) algorithms based on artificial neural networks (Kihm et al., 2018). New optical concepts using optofluidic microlenses-like behavior of RBCs (Mugnano et al., 2018) and indirect adaptive optics as well as label-free quantitative phase imaging (Miccio et al., 2015) enables assessment of cell volume of individual cells, and monitoring of morphometric features (e.g., label-free optical markers) that make high throughput reliable quantification of cell phenotypes possible. It allows to stay unbiased, omit “human factor,” and allocate RBC shapes to a continuous scale with high throughput and precision. The challenge is that artificial neural networks need to be set up, customized and most notably trained. This type of analysis will become available routinely in the nearest future.

### Hydration State and Density

**FIGURE 1:**
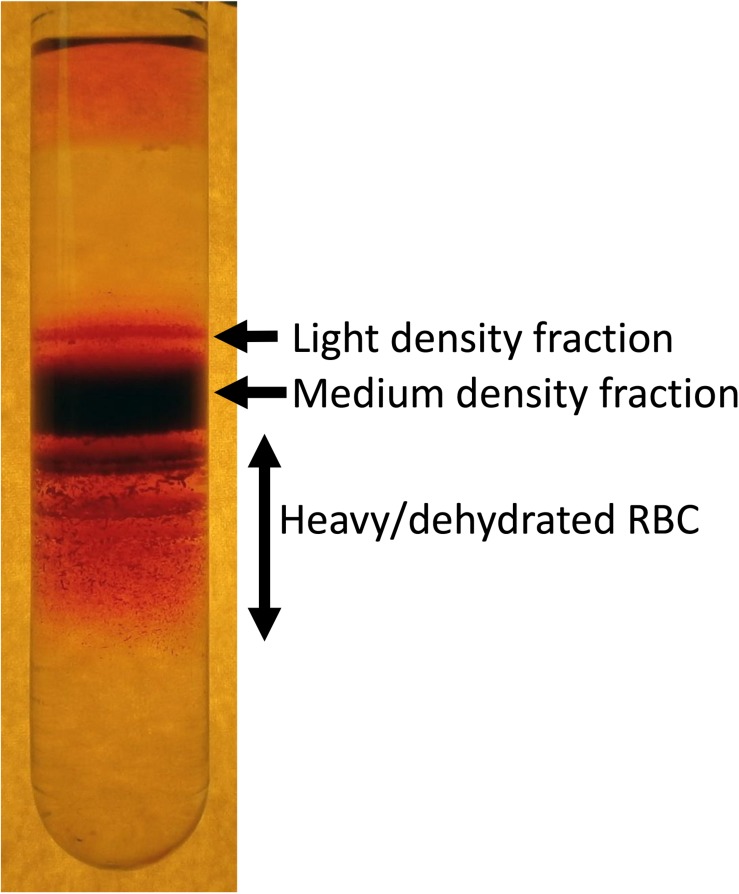
An example of heterogeneity of RBC density revealed by fractionation of RBCs on a self-forming Percoll density gradient. Composition of light, medium and high-density fractions vary depending on human health and environmental stress. About 15–20% of RBCs of healthy human donors forming low density are reticulocytes. However, along with young cells this fraction is “contaminated” with swollen RBCs at the terminal senescence stage (Lew and Tiffert, 2013). Medium fraction is formed by mature RBCs, and heavy dehydrated cells are those with senescent phenotype.

The best method to visualize the variance in RBC density is fractionation on a Percoll (Figure 1), Ficoll, Stractan, or phtalate density gradient (Danon and Marikovsky, 1964; Corry et al., 1982; Salvo et al., 1982; Mosca et al., 1991; Lutz et al., 1992). Upon centrifugation in isotonic solution of any of these materials forming continuous or discontinuous gradients, RBCs distribute within them according to their densities. As RBCs of healthy human donors are fractionated on a self-forming Percoll gradient, three to five fractions may be collected. A small fraction of cells with lower density bands as the top layer, followed by one or several RBC populations with a medium density and a minor fraction of cells is presented with the highest density (Lutz et al., 1992; Makhro et al., 2013; Makhro et al., 2016b). RBCs of patients with hereditary hemolytic anemias are generally characterized with a broader variance in densities. Often this diversity may contain clinically relevant information on the severity of disease state. For sickle cell disease the abundance of dense cells was suggested to be a predictor of severity of disease manifestation due to the increased probability of irreversible aggregation of HbS (Kaul et al., 1983). For hereditary spherocytosis severity is associated with an increase in abundance of well-hydrated cells that are lost before they have time to mature and lose some of their membrane (Huisjes et al., 2019). Increase in heterogeneity is high in patients with cryohydrocytosis (Bogdanova et al., 2010), Gardos channelopathy (Fermo et al., 2017) beta-thalassemia, G6PD, and pyruvate kinase (PK) deficiency (Mosca et al., 1991).

Factors defining RBC density include changes in water and ion content and membrane loss. During the density fractionation RBCs experience shear stress during centrifugation as they move through the isotonic Percoll solution containing micromolar concentrations of Ca^2+^ in the absence of EGTA. Shear forces may activate mechano-sensitive channels such as PIEZO1 channels (Cahalan et al., 2015) and NMDA receptors (Hanggi et al., 2014) that are permeable for Ca^2+^. Uptake of Ca^2+^ via these receptors triggers loss of K^+^ mediated by opening of Ca^2+^-dependent Gardos channels. Thus, fractionation of RBCs on Percoll should be viewed as a functional test in which distribution of the cells is not only driven by the steady state density, but also by their mechano-sensitivity.

Indirect methods to assess heterogeneity in RBC density include detection of hypo- and hyperchromic cells in blood smears, HDW as well as the shape of the curve in osmotic fragility test of the right arm of the osmoscan curve obtained by ektacytometry (Clark et al., 1983; Lutz et al., 1992). Hight throughput devices for evaluation of RBC density using functional tests at the single cell level are being developed.

### Ca^2+^ Levels (Static and Dynamic Tests) and Electrophysiological Properties

Heterogeneity in basal free Ca^2+^ levels was recorded in RBCs of healthy humans (Kaestner et al., 2006; Makhro et al., 2013; Fermo et al., 2017). Stimulation of Ca^2+^ uptake by treatment of healthy human RBCs with PGE_2_ (Danielczok et al., 2017), lysophosphatidic acid (Steffen et al., 2011; Kaestner et al., 2012; Wang et al., 2013; Wesseling et al., 2016) or glutamate (Makhro et al., 2013; Hanggi et al., 2014; Makhro et al., 2016a; Petkova-Kirova et al., 2019) increases variance in the intracellular Ca^2+^. Not all cells respond to shear stress or pro-oxidative condition with an increase in Ca^2+^.

Molecular causes for this heterogeneity in responses to various stressors are poorly understood. It is obvious, that they relate to the differences in abundance of either Ca^2+^ channels (Kaestner et al., 1999; Makhro et al., 2013; Kaestner and Egee, 2018; Rotordam et al., 2019) or of the primary receptors responding to the stressor (such as LPA or prostaglandin receptors; Wang et al., 2013; Danielczok et al., 2017). In human RBCs several ion channels are known to mediate Ca^2+^ uptake including PIEZO1, TRPC6, NMDA receptors, Ca_V_2.1 and several others (for a recent review see Kaestner et al., 2020). As a result of stochastic distribution and opening probability, Ca^2+^ entry into individual RBCs varies in response to stimulation by individual Ca^2+^ channels substantially giving rise to “responders” and “non-responders” cellular sup-populations. This uneven behavior may be further amplified due to the existence of feedback loops supporting Ca^2+^-dependent Ca^2+^ uptake (Kaestner et al., 2018).

Most documented is inter-cellular variance in distribution of the Ca^2+^-dependent K^+^ (Gardos) channel in RBCs. However, majority of the recordings for this best-studied channel in RBCs were performed as mean values for the unseparated populations, using radioactive tracer kinetics technique or single channel recordings. Reports based on whole-cell recordings for this channel are still sparse (Kucherenko et al., 2005; Kucherenko et al., 2013; Fermo et al., 2017). A further factor that may amplify heterogeneity of Gardos channel recordings in RBCs is its inactivation upon hypoxic exercises (Mao et al., 2011).

### Redox State and Metabolism

Staining of individual cells with fluorescent probes sensitive to pro-oxidative free radicals such as dicarbofluorescein (Amer et al., 2003; Grinberg et al., 2005) and monobromobimane (Kosower and Kosower, 1995) provide a possibility to follow the changes in redox balance in individual cells. One more approach to record redox state in sub-fractions of RBCs is based on pre-fractionation of cells into low, medium and high density fractions before assessment of reduced and oxidized glutathione (GSH and GSSG) and NAD(P)H (Piccinini et al., 1995; D’Alessandro et al., 2013) in these sub-populations. Dense cells were shown to be deprived of GSH and enriched with GSSG compared to the mature RBCs of medium density. Accumulation of GSSG and reduction in GSH was not associated with any substantial changes in the intracellular ATP or NADPH (Sass et al., 1965; D’Alessandro et al., 2013). Finally, redox state of RBCs may be expressed as the ability to tolerate oxidative challenge (Lisovskaya et al., 2008; Sinha et al., 2015) which differs between individual RBCs as well.

Shifts in redox equilibrium in RBCs of healthy donors are associated with age-dependent decrease in pyruvate kinase, hexokinase, glucose-phosphate dehydrogenase, aldolase activities (Salvo et al., 1982; Suzuki and Dale, 1988). Oxidative stress is a hallmark of RBCs of patients with hereditary hemolytic anemias presented with one or two alleles of mutated glucose-6 phosphate dehydrogenase (G6PD). The resulting in acute hemolytic condition known as favism is associated with depletion in NADPH in favor of NADP^+^ (Mason et al., 2007; Peters and van Noorden, 2017). Furthermore, systemic oxidative stress caused by inflammatory processes, infection and other causes may result in release of reduced glutathione from RBCs and temporary increase in oxidative load and aggravate the differences in redox state between the cells of different ages (Giustarini et al., 2008).

### Hb Levels and Variants

Inter-cellular heterogeneity in intracellular hemoglobin content in clinical settings is reflected by the abundance of hypochromic and hyperchromic cells in blood smears. The abundance of hypochrome RBCs for healthy humans should not exceed 2.5% of circulating RBCs (Macdougall et al., 1992; Schaefer and Schaefer, 1995; Braun et al., 1997), dropping below 1% in patients with iron overload, and increasing to 20% and more in patients with iron deficiency. Higher levels of hyperchromic cells was also reported for patients with hereditary spherocytosis (Conway et al., 2002) and sickle cell disease (Ballas and Kocher, 1988).

Even more intercellular heterogeneity is introduced by a pronounced variance in the presence of fetal hemoglobin in a small fraction of cells (F-cells) in healthy humans (Boyer et al., 1975; Thein and Craig, 1998). The abundance of F-cells increases during high altitude exposure (Narayan et al., 2005). Pregnancy has an impact on this parameter (Prus and Fibach, 2013). Moreover, the abundance of F-cells as well as the amount of HbF in them may differ from cell to cell in patients with beta-thalassemia (Narayan et al., 2005). In sickle cell disease, HbF abundance furthermore strongly depends on the haplotype (Menzel and Thein, 2019). Sickle cell trait results in uneven distribution of HbS between the cells, and the pattern for such variance seems to be hereditary (Anyaibe et al., 1985).

## Causes of Heterogeneity

If we want to make extensive use of RBC heterogeneity as a diagnostic and prognostic marker, we have to understand the origin of the observed variance in RBC properties.

This may stem from the different pools of erythroid precursor cells that equip the resulting reticulocytes with various sets of proteins that may only be produced as long as the synthesis machinery is active before the enucleation.

The other cause of heterogeneity are the age-dependent differences between the young, mature and senescent RBCs. The third cause occurring at the systemic level originates from the alteration in the micro- and macro-environmental conditions (changes in hormonal and metabolic levels, inflammation, shear stress load, hyperthermia and others).

These three sources of heterogeneity will be reviewed below.

### Cellular Heterogeneity During Erythropoiesis

**FIGURE 2:**
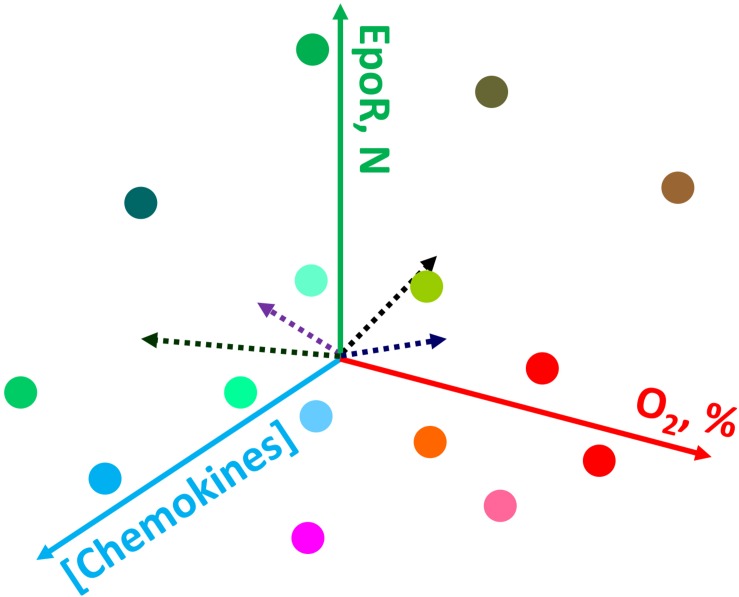
Schematic representation of the possible causes of heterogeneity for erythroid precursor cells within bone marrow. Gradients in oxygen availability, chemokines and other signaling messengers create a plethora of conditions in which cells find themselves during differentiation. For details see the text.

Accumulated evidence over the last 25 years has demonstrated the existence of heterogeneity within the erythroid compartment schematically shown in Figure 2. Although it is quite expected to have a heterogenic population within the mature RBC population due to the long- life span of mature RBCs (100–120 days in human) representing cells of various ages, it is less clear and more intriguing the reasons for erythroid precursors/progenitors to be heterogeneous in multiple facets of their form and function.

One of the most established and well-explained aspect of erythroid precursor heterogeneity pertains to erythroid precursors possessing differing sensitivities to erythropoietin (EPO). Soon after discovering the precise molecular function of EPO to be a cell survival function (Koury and Bondurant, 1988, 1990), studies revealed that even within a highly homogenous population in terms of the differentiation stage (operationally defined as colony-forming unit-erythroid; CFU-E), erythroid precursors underwent apoptosis following EPO withdrawal in an asynchronous manner (Kelley et al., 1993). These studies demonstrated a dose response effect as reflected by increasing numbers of CFU-Es undergoing apoptosis as EPO concentrations were gradually decreased. These observations clearly highlighted the built-in heterogeneity within the developing erythroid cell compartment with respect to the biochemical nature of each cell within an otherwise “homogenous” precursor pool as defined by morphological characteristics. One of the possible causes supporting heterogeneity are the gradients in various signaling messengers, growth factors, chemokines, oxygen levels and the resulting reactive oxygen species, and other factors (e.g., Thompson et al., 2010; Spencer et al., 2014; Itkin et al., 2016) making conditions in which precursor cells differentiate unique and dependent on their location within the bone marrow (Figure 2). An elegant model proposed by Koury and Bondurant (1992), explained the basis of differing EPO sensitivities as a built-in mechanism to prevent all erythroid precursors undergoing apoptosis during low EPO levels in circulation such as in patients with renal failure. The work by several other groups (Miura et al., 1991; Landschulz et al., 1992; Nakamura et al., 1992; Kelley et al., 1993) had shown that heterogeneic EPO response within the same precursor population cannot be attributed to the numbers of EPO-receptors, affinity or structure, thereby suggesting differences in signal transduction as the likely mechanism for the existence of heterogeneity in EPO response. Based on these findings one can appreciate the existence of signaling heterogeneity within the erythroid precursor compartment as a necessary component during the development process to yield mature red blood cells. Recently developed single-cell intracellular flow cytometry approaches (Liu et al., 2019) are bound to further uncover previously unrecognized levels of regulatory heterogeneity during erythroid cell development.

Besides the existence of biochemical/signaling heterogeneity within the developing erythroid precursors other aspects of erythroid precursor heterogeneity have been observed especially most recently due to the advancement of single-cell technologies at both trascriptomic and phenotypic levels (Woll et al., 2014; La Manno et al., 2018; Brierley and Mead, 2019). Within the erythroid compartment especially during the early stages of erythropoiesis a significant level of transcriptomic variability and heterogeneity seem to exist at least based on mouse bone marrow erythroid precursors (Tusi et al., 2018). The same study also found that cell cycle in erythroid precursors are continuously remodeled during the differentiation program but consistent with very early studies using bulk erythroid precursors (CFU-E), the vast majority of cells were in the S-phase of the cell cycle (Iscove, 1977). These results demonstrate that an individual cell, especially during development, has the ability to program itself to act not in concert each other with respect to signal transduction, gene transcription, cell cycle and many other aspects even though morphologically a cell population may look alike at a particular stage of differentiation.

Overall, accumulated data suggests that heterogeneity during erythroid development may not be evenly spread during the entire development cascade. Most data points to greatest level of inter-cellular heterogeneity during the early phases of development when these cells are responsive to various growth factors. Beyond the late polychromatic stage, when the cells have exited the cell cycle one observes less heterogeneity and most cells undergo dramatic reduction in cell size, chromatin condensation and enucleation. However, it is conceivable even in the bone marrow niche within the blood island not all erythroblasts undergo enucleation adding another layer of heterogeneity. It is also conceivable that due to differing levels of chemokine receptors on these cells the progenitors also exhibit varying degrees of migration within the bone marrow niche. Overall, it may seem the inter-cellular heterogeneity during erythroid precursor development. Each cell is possessing different sensitivity to EPO, and as a result a vast majority of precursors die due to apoptosis, the strategy that seems quite wasteful. However, we speculate that such heterogeneity is critical in order to respond to rapid changes in the micro and macro environment such as changes in oxygen concentration due to changes in altitude, pro-inflammatory and oxidative stress conditions as well as sudden blood loss due to trauma and onset of anemia due to renal failure.

### Age of RBCs

**FIGURE 3:**
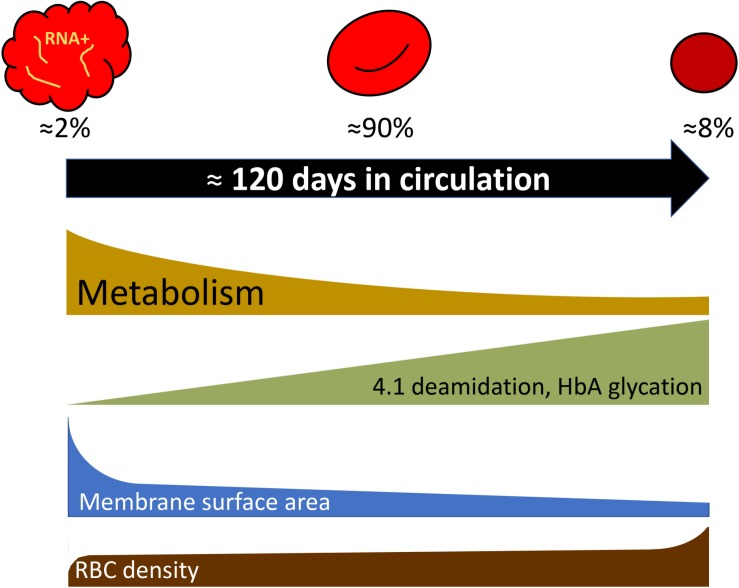
Selected parameters that change during RBC aging following exponential or linear kinetics as cells turn from reticulocytes to mature cells and finally enter the senescent stage. Percentage of reticulocytes, mature cells, and dense senescent cells shown in the scheme correspond to those in adult healthy donors. For more details see Lutz and Bogdanova (2013) and the text.

Most of the findings for the age-related variance for RBCs of healthy humans were obtained for the fractions of cells of low, medium and high density, that were enriched with young, mature and senescent cells, respectively (Mueller et al., 1985, 1987; Lutz et al., 1992; Figure 1). Gradual changes occurring with cell aging were described in several reviews (Lutz and Bogdanova, 2013; Lew and Tiffert, 2017; Badior and Casey, 2018; Minetti et al., 2018) and article collections (Beutler, 1988; Mangani, 1991), and schematically represented in Figure 3.

Recent studies of the age-dependent changes in RBCs involve single cell approaches such as flow cytometry and microscopy as well as proteomics (D’Alessandro et al., 2013; Minetti et al., 2013).

Deamidation of asparagine residue 502 of the band 4.1 protein was shown to occur gradually with RBC age as the deamidation rate is an exclusive function of temperature and time (Inaba and Maede, 1988, 1992). Deamidation is manifested as an appearance of a double band on the gels as the native and deamidated form of the protein differ in electrophoretic mobility of the protein. Fractionation of RBCs of healthy humans according to their density has shown that young cells have lower density than mature cells. Senescence is associated with further increase in RBC density and mean corpuscular hemoglobin concentration, and reduction in RBC volume. Using the changes in deamidation of band 4.1 protein or direct labeling of RBCs and monitoring of their aging (Luthra et al., 1979), increase in density were revealed as an intrinsic feature of *in vivo* aging of RBCs of healthy humans. Dense cells obtained by fractionation of leukodepleted RBCs on Percoll density gradient were presented with substantially lower GSH levels and GSSG levels that were doubled compared to the mature RBCs, whereas ATP and NADPH levels were only slightly reduced in the densest cell fractions (Sass et al., 1965; D’Alessandro et al., 2013). These changes were associated with the age-driven decrease in pyruvate kinase, hexokinase, glucose-6-phosphate dehydrogenase, aldolase activities (Salvo et al., 1982; Suzuki and Dale, 1988). Some of the terminally senescent RBCs, that lose control over their Na^+^ gradients and volume regulation due to the reduction in Na,K-ATPase activity, were reported to swell and lyse (Lew and Tiffert, 2013, 2017).

Reports on the changes in free Ca^2+^ levels are controversial and depend on the techniques used for assessment of these parameters (Romero and Romero, 1997, 1999; Makhro et al., 2013; Lew and Tiffert, 2017). Both Ca^2+^-permeable channel activity and that of plasma membrane Ca^2+^ pumps decreases with cellular aging (Romero et al., 2002; Makhro et al., 2013). Despite this inconsistency, changes in the intracellular free Ca^2+^ and the ability to maintain low levels of Ca^2+^ are the factors in control of RBC longevity (Bogdanova et al., 2013; Lew and Tiffert, 2017).

Further hallmarks of RBC aging include the changes in phosphorylation pattern (Fairbanks et al., 1983) and membrane loss (Mohandas and Groner, 1989).

### Physical Activity, High Altitude, and Other Stress Conditions

How substantial would the change be at the level of circulating RBCs if the gene expression reprogramming occurs at the level of precursor cells? Simple calculations assuming that the RBC longevity is not affected by these changes and all cells are equally affected by this change, gives a rough estimate of ∼0.82% of RBC population changing per day for the “normal” production rate of 2.4 × 10^6^ cells/s. If erythropoiesis is boosted to its maximum (10-fold increase, 8.2% of new cells will appear daily (Elliott and Molineux, 2009). This means that acute reversible changes at the bone marrow level will hardly be noticed if stress conditions persist for just 24 h. On the contrary, when stress conditions boosting erythropoiesis persist for a week, 5.7–57% of cells will get a new feature.

**FIGURE 4:**
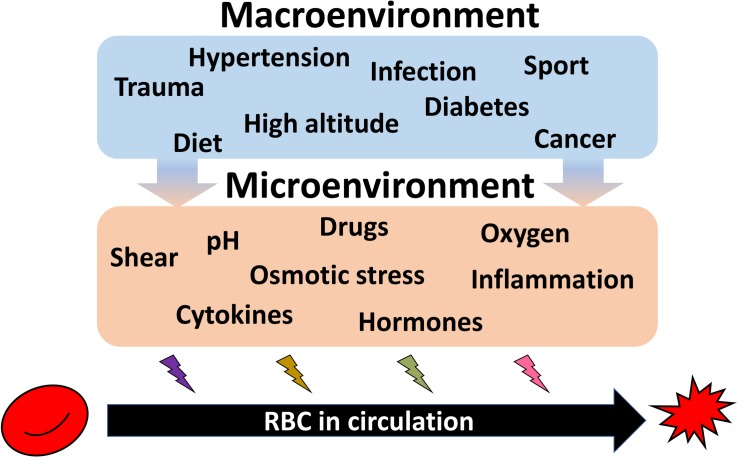
Summary on the environmental causes imposing heterogeneity of circulating RBCs. Exposure of the organism to high altitude or practicing endurance sport as well as dietary preferences cause durable or acute impact on the RBC properties. Along with RBC diseases (anemia, polycythemia), pathologies such as hypertension, diabetes, infection, trauma, cancer, and further systemic diseases are influencing both erythropoietic niche and the circulating cells. All these macroenvironmental stresses translated into the changes in microenvironment for erythroid precursors and circulating RBCs. Shear, alterations in pH and oxygen levels, proinflammatory cytokines, and hormones, as well as drugs work to shape the features of each individual RBC resulting in an increase in the inter-cellular heterogeneity.

Such kinetics does not favor *de novo* production as an efficient strategy for acute adaptation to hypoxia or single endurance sport exercise bout, dietary changes, or to pathological conditions such as infection or sepsis, cancer, diabetes, or cardiovascular diseases (Figure 4). These changes in turn translate into the changes in shear stress, oxygen availability, pH, hormones and proinflammatory cytokines and other microenvironmental factors sensed by RBC directly. Species that undergo such acute changes from hyperoxygenation to severe hypoxia, such as Rainbow trout (*Oncorhynchus mykiss*) (Fago et al., 2001) or Rüppell’s griffon vulture (*Gyps rueppelli*). Rüppell’s griffon vulture was spotted at 37,000 feet (11277.6 m) when colliding with the plane (Laybourne, 1974) permanently possess several hemoglobin variants. Hemoglobin A and D chains are present in RBC vulture producing high and low affinity hemoglobin variants and allowing these unique birds to fly above 10,000 m with no need to engage any complex adaptive processes as they land (Weber et al., 1988; Hiebl et al., 1989).

Adult humans have by far lower adaptive capacity, possessing generally one Hb variant, HbA with some minor additions of HbF. However, plasticity of O_2_ delivery, and its fast on-demand optimization upon the changes in environmental O_2_ availability may be associated with other types of heterogeneity in RBC structure and function. Potential adaptive role of variance in RBC properties has to be further explored.

It is largely accepted that multiple forms of pathologies, both related to abnormal structure RBC membrane or cytosolic proteins and lipids, as well as systemic disorders such as cancer, diabetes, cardiovascular diseases, sepsis and other diseases of inflammation are associated with anemia, RBC damage and their premature removal from the circulation and increase in their heterogeneity (e.g., Salvagno et al., 2015; Feng et al., 2017; Ahmad et al., 2018; Ko et al., 2018; Yin et al., 2018; Parizadeh et al., 2019; Wang et al., 2019). The causes and consequences as well as predictive power of this increase in variability of RBC properties is out of the scope of this review but deserve special attention.

## Summary and the Standing Challenges

The present collection of information on the possible causes and consequences of inter-cellular heterogeneity justifies the increasing attention of researchers to the RBC sub-populations and individual cells. It appears that vast amount of information on the near and distant (within months) past is lost when RBC properties are reduced to a set of single “mean” values. This information appears to be of substantial importance when severity of disease or efficacy of therapy are to be assessed for individual patients. At present we do not have the commercially available and standardized methodologies and machines to be able to compare the data obtained of the single cell features in different labs. These challenges are already addressed by some researchers and will drive the transformation of our understanding of red blood cell biology in the nearest future.

## Author Contributions

AB and AW have composed the text. All authors contributed to editing and proofreading of the text.

## Conflict of Interest

The authors declare that the research was conducted in the absence of any commercial or financial relationships that could be construed as a potential conflict of interest.
